# Development and Morphological/Microstructural Characterization of a Novel Synergic Synbiotic Co‐Encapsulated *Lactobacillus* spp. Consortium and Kale Powder

**DOI:** 10.1002/fsn3.71314

**Published:** 2025-12-03

**Authors:** Monica Villanueva‐Castañeda, Mayra Antunez‐Mojica, Daniel Tapia‐Maruri, Ollin Celeste Martínez Ramírez, Raúl Dávila Delgado, América Ivette Barrera‐Molina

**Affiliations:** ^1^ Facultad de Nutrición Universidad Autónoma del Estado de Morelos Cuernavaca Mexico; ^2^ SECIHTI‐Centro de Investigaciones Químicas‐IICBA Universidad Autónoma del Estado de Morelos Cuernavaca Mexico; ^3^ Centro de Desarrollo Productos Bióticos, Instituto Politécnico Nacional San Isidro Mexico

**Keywords:** co‐encapsulation, kale, *Lactobacillus*, polyphenols, prebiotic, probiotic, symbiotic

## Abstract

Kale, a nutrient‐dense leafy green rich in bioactive compounds, has emerged as a promising prebiotic candidate for enhancing probiotic viability and functionality. This study investigated the effects of kale powder and its polyphenolic compounds on the growth and stability of a *Lactobacillus* spp. consortium. Growth kinetics experiments revealed that 1% kale powder significantly enhanced bacterial growth, with a pronounced increase observed after 5 h of incubation compared to the control. Furthermore, a synergistic synbiotic was successfully co‐encapsulated using a 1.3% alginate‐based matrix combined with 1% kale powder and *Lactobacillus* spp. consortium. Morphological characterization under varying temperature conditions demonstrated that co‐encapsulated particles maintained structural integrity at 4°C and during freezing, while dehydration led to significant size reduction due to moisture loss. Encapsulation efficiency reached 93%, with the alginate matrix effectively protecting bacterial viability, as evidenced by only a 13% reduction in viability after storage at 4°C. Microscopic analyses confirmed the presence of polyphenols within the co‐encapsulated system, with confocal microscopy revealing distinct autofluorescence attributed to phenolic compounds. Electron microscopy further showed that co‐encapsulated particles remained intact under refrigeration and freezing but exhibited morphological changes after dehydration. Growth kinetics of the *Lactobacillus* spp. consortium in medium supplemented with kale‐derived polyphenols indicated optimal growth at 0.4% concentration, while higher concentrations (0.6%) led to reduced growth, suggesting substrate inhibition. These findings highlight the dual role of kale polyphenols as prebiotic substrates and protective agents, underscoring their potential in developing stable and functional synbiotic delivery systems for probiotic applications.

## Graphical abstract

A co‐encapsulated synbiotic of *Lactobacillus* spp. and kale powder was developed using a 1.3% alginate matrix, achieving 93% encapsulation efficiency. Kale at 1% significantly enhanced bacterial growth. Polyphenols acted as both prebiotics and protective agents, preserving > 85% viability after storage at 4°C. Particles retained structural integrity under refrigeration and freezing but shrank upon dehydration. Optimal growth occurred at 0.4% polyphenol concentration; higher levels caused inhibition. These findings confirm the dual potential of kale in developing stable, functional synbiotic systems for probiotic applications.

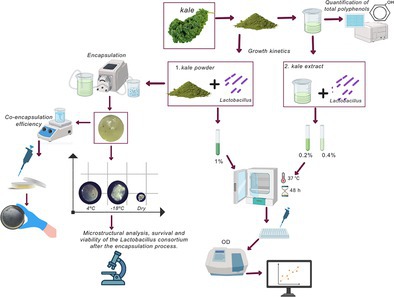

## 1 Introduction

Current global trends indicate an increased use of probiotics, their products (postbiotics) and prebiotics to alleviate and prevent various infections and non‐infectious diseases, including gastrointestinal and cardiovascular conditions. Probiotics are live, well‐defined, and viable microorganisms that, in sufficient quantities, benefit host health by modifying the composition of the intestinal microbiota (Kerry et al. 2018; FAO/WHO 2001; Ji et al. 2023). Among their benefits are postnatal intestinal maturation, immune modulation, mucosal barrier fortification, enhanced absorption of macronutrients and micronutrients by metabolizing nutritional components that the host cannot process on its own, xenobiotic metabolism and others (Yadav et al. 2022). *Lactobacillus* spp. are among the most commonly used bacteria as probiotics due to their beneficial effects on gut microbiota balance and stability. Different strains of *Lactobacillus* used individually or in consortium can stabilize the intestinal microbiota while stimulating, modulating, and regulating the immune response (Yadav et al. 2022). These actions positively influence the host's metabolism by improving weight management, reducing body fat mass, and lowering the risk of metabolic syndrome (Hamdan et al. 2016; Minami et al. 2018; Andreasen et al. 2010). However, the intake of probiotics alone does not guarantee bacterial survival, growth, and colonization. To enhance these processes, probiotics are combined with nondigestible food ingredients, known as prebiotics, which selectively stimulate the growth and/or activity of specific bacteria in the colon, thereby improving host health (Gibson and Roberfroid 1995; Yadav et al. 2022). Carbohydrates are typically the primary and best‐characterized sources of prebiotics. There is growing interest in identifying new prebiotic candidates derived from non‐carbohydrate substances, such as short‐chain fatty acids and polyphenols (Cunningham et al. 2021). Polyphenols, have garnered significant attention due to their well‐documented antioxidant properties, which play a role in preventing and managing oxidative stress‐related diseases, among other health benefits (Kumar, Kumar, et al. 2023; Kumar, Debnath, and Singh 2023). However, it is estimated that 90%–95% of dietary polyphenols are not absorbed in the small intestine and, therefore, reach the colon, where they are likely metabolized by the microorganisms of the gut microbiota (Cunningham et al. 2021). Plant‐derived metabolites are excellent sources of polyphenolic compounds. Kale ( *Brassica oleracea* var. *sabellica*) is a cruciferous vegetable from the Brassicaceae family, increasingly used in food due to its nutritional characteristics, including a high content of vitamins A, C, E, and K, minerals such as iron, calcium, and vitamins like folate, as well as its fiber and polyphenolic compounds (Valencia, Choque, and Mamani 2022; Valencia, Cardona, and García 2022; Cardona, García, and Mc 2013; Cardona, Andrés‐Lacueva, et al. 2013). The co‐administration of probiotics and prebiotics (synbiotics) is an effective strategy to enhance bacterial survival and improve health benefits for the host. Common administration methods include capsules, tablets, sachets, beverages, and other delivery formats (Swanson et al. 2020). Co‐encapsulation in protective matrices offers several advantages, including preserving the viability of probiotics during processing and storage, as well as ensuring their effectiveness in the gastrointestinal tract (López and Michiutti 2019). Similarly, bioactive compounds, when encapsulated, are protected from degradation, optimizing their absorption in the body (Ducatelle et al. 2014; Quigley et al. 2017). The success of these co‐encapsulation systems, however, is fundamentally linked to their morphological and microstructural properties—such as particle size, surface topography, and structural integrity—which directly influence the stability, protection, and release of the core components. These systems enhance the beneficial effects of probiotics and bioactive compounds, enabling their incorporation into functional foods and supplements, promoting improved intestinal and overall health. This study aimed to characterize the morphology and microstructure of a novel synbiotic co‐encapsulation system containing a *Lactobacillus* spp. consortium and kale powder.

## 2 Materials and Methods

### 2.1 Bacterial Strains and Activation Conditions

The strains *Lactobacillus delbrueckii* subsp. bulgaricus and *Lactobacillus delbrueckii* subsp. *lactis* were obtained together as a single, commercial lyophilized consortium (Danisco France SA). These bacterial strains were activated in MRS broth culture medium (Man, Rogosa, and Sharpe, BD Bioxon brand) at 37°C, under microaerophilic conditions, with 5% CO_2_ for 24 h. After the strains were activated, bacterial growth was performed using the same activation conditions.

### 2.2 Growth Conditions of *Lactobacillus* spp. Consortium in the Presence of Kale Powder

To determine the prebiotic effect of kale powder on *Lactobacillus* spp. consortium, growth kinetics were assessed by inoculating 500 μL of a fresh culture into 50 mL of MRS broth culture medium, previously supplemented with kale powder at 1%. The cultures were incubated for 48 h under anaerobic conditions and 200 μL aliquots were taken at 2‐, 4‐, 6‐, 8‐, 24‐, and 48‐h *post*‐incubation. The optical density (OD) was measured at 620 nm using a Labsystems Multiskan Ascent 354 microtiter plate reader. Negative controls included MRS broth, and MRS with the *Lactobacillus* spp. consortium only.

### 2.3 Co‐Encapsulation of *Lactobacillus* spp. Consortium and Kale Powder

Microcapsules were developed using sodium alginate with high guluronic acid content (COSMOPOLITA brand, food‐grade). This alginate was specifically selected due to its ability to form a robust ‘egg‐box’ matrix with calcium ions, providing effective protection for the *Lactobacillus* consortium and kale powder. Food‐grade calcium chloride was used for gelation. Three different alginate/kale concentration combinations were tested to optimize the encapsulation system: (I) sodium alginate matrix at 1.3%, (II) matrix with kale powder, and (III) matrix with kale powder and *Lactobacillus* spp. consortium, aiming to optimize consistency and functionality. An aqueous solution of 1% (w/v) kale powder was prepared and homogenized by agitation for 15 min, followed by the addition of 500 μL of *Lactobacillus* spp. consortium suspension (6 × 10^8^ CFU/mL) per 10 mL of solution. Sodium alginate (1.3% w/v) was then slowly incorporated under gentle agitation at 35°C for 3 min. The mixture was introduced into a 5% (w/v) calcium chloride (CaCl₂) solution using an INTELLAB peristaltic pump (100–240 V, 50 Hz/60 Hz) at a feed rate of 5 mL/min, forming microcapsules via crosslinking. After formation, the microcapsules were washed with distilled water to remove excess CaCl₂, transferred into 50 mL Falcon tubes, and stored under refrigeration at 4°C, freezing or drying to assess stability.

### 2.4 Co‐Encapsulation Efficiency Assay of *Lactobacillus* spp. Consortium With Kale Powder

To estimate the co‐encapsulation efficiency and survival of the *Lactobacillus* spp. consortium, plate count methods were employed before and after encapsulation, following protocols adapted from López‐Fernández et al. (2019) and Michiutti and Hernan (2019). Co‐encapsulated samples (4 g) were dissolved in 10 mL of MRS broth enriched with 5% sodium citrate, followed by agitation at 37°C for 30 min to release the bacteria. Serial dilutions of the dissolved samples were prepared, and 100 μL of each dilution was spread onto Petri dishes containing MRS‐agar. The plates were incubated at 37°C for 24 h, after which colony‐forming units (CFU) were counted. Co‐encapsulation efficiency was determined using the following calculation:

$$\text{Encapsulation Efficiency}\;\left(\%\right)=\left(\frac{\mathrm{CFU}\;\text{after encapsulation}}{\mathrm{CFU}\;\text{before encapsulation}}\right)\times 100$$

### 2.5 Morphological Characterization of Co‐Encapsulates

The morphological characterization of the co‐encapsulates was performed through detailed image analysis. Digital images were captured using an Olympus XM10 CCD camera coupled to an Olympus MVX10 stereomicroscope equipped with a 1× objective and a white light illumination system. Images were saved in TIFF format with a resolution of 1024 × 768 pixels for subsequent analysis. The diameter of the co‐encapsulates was quantified using the Feret diameter tool in ImageJ software (https://imagej.nih.gov/ij/index.html), which provided precise particle size measurements. Additionally, the stability of the co‐encapsulates was evaluated under three storage conditions: refrigeration (4°C), freezing (−18°C), and dehydration. This comprehensive analysis allowed for the assessment of structural integrity and morphological changes under varying environmental conditions, providing insights into the robustness of the encapsulation system.

### 2.6 Microstructural and Survival Analysis of Integrated Particles of Kale Powder and *Lactobacillus* spp.

Microstructural characterization of the samples was conducted using electron and confocal microscopy techniques. For electron microscopy, samples were mounted on aluminum stubs using double‐sided carbon conductive tape and analyzed using an environmental scanning electron microscope (Carl Zeiss, EVO LS10, Germany). Observations were performed at an accelerating voltage of 15 kV and a chamber pressure of 70 Pa. A backscattered electron detector (NTS BSD) was employed to capture high‐resolution images, which were saved in grayscale TIFF format with a resolution of 1024 × 768 pixels. For confocal microscopy, samples were placed on glass slides and examined using a CLSM microscope (Carl Zeiss, LSM800, Germany) equipped with a color AxioCam HD camera (Carl Zeiss, Model 305, Germany). The system was controlled by ZEN (Zeiss Efficient Navigation) software, version 2.6 blue edition. Micrographs were acquired using 5× and 20× apochromatic objectives with numerical apertures of 0.8 and 1.3, respectively. All images were stored in TIFF format at 2048 × 2048 pixels. The “lambda mode” tool was utilized to detect autofluorescence in the samples, employing solid‐state lasers at 488 nm (green) and 640 nm (red) with 5% excitation and a pinhole setting of 0.8 Airy Units (AU). Additionally, viability assessment was performed using Acridine Orange and Propidium Iodide III counterstains to differentiate between viable and nonviable cells.

### 2.7 Preparation of Extracts and Quantification of Total Polyphenols

Phenolic compounds were quantified from kale powder (Euphoria brand) using aqueous extraction. A 1% (w/v) kale solution was prepared by dissolving 25 g of kale powder in water, stirring the mixture for 2 h at room temperature, followed by vacuum‐filtering, and subsequently lyophilizing to obtain a dry extract for analysis.

The total phenolic compounds of the aqueous extract were quantified by the Folin–Ciocalteu method (Teixeira et al. 2016). Folin–Ciocalteu reagent was used at 2 N and diluted 1:2 with water, and a NaHCO_3_ solution (20% w/v), was incubated in the dark for 30 min. The absorbance was measured at 760 nm at a UV/Vis microplate spectrophotometer, Multiskan. The total phenolic content was calculated using a calibration curve of gallic acid, and the result was expressed as gallic acid equivalent (mg GAE/100 g).

### 2.8 Growth Evaluation of *Lactobacillus* spp. in Aqueous Kale Extract

Growth kinetics experiments were conducted using an aqueous kale extract to assess the prebiotic potential of kale powder. MRS broth solutions were prepared and supplemented with varying concentrations of the aqueous kale extract (0.2% and 0.4%). To each solution, 500 μL of a previously activated *Lactobacillus* spp. consortium was added. The growth kinetics were monitored over a 48‐h period, following the methodology described in previous sections.

### 2.9 Statistical Analyses

To ensure the reproducibility of the results, statistical analysis was conducted on data obtained from three biological replicates using GraphPad Prism software (version 10). Differences between values at various incubation times in time‐course experiments were evaluated using one‐way ANOVA, followed by Tukey's multiple comparisons test for post hoc analysis. Statistical significance was determined based on *p* < 0.05 indicated in the corresponding figure legends.

## 3 Results

### 3.1 Effect of Kale on the Growth of *Lactobacillus* spp.

**FIGURE 1:**
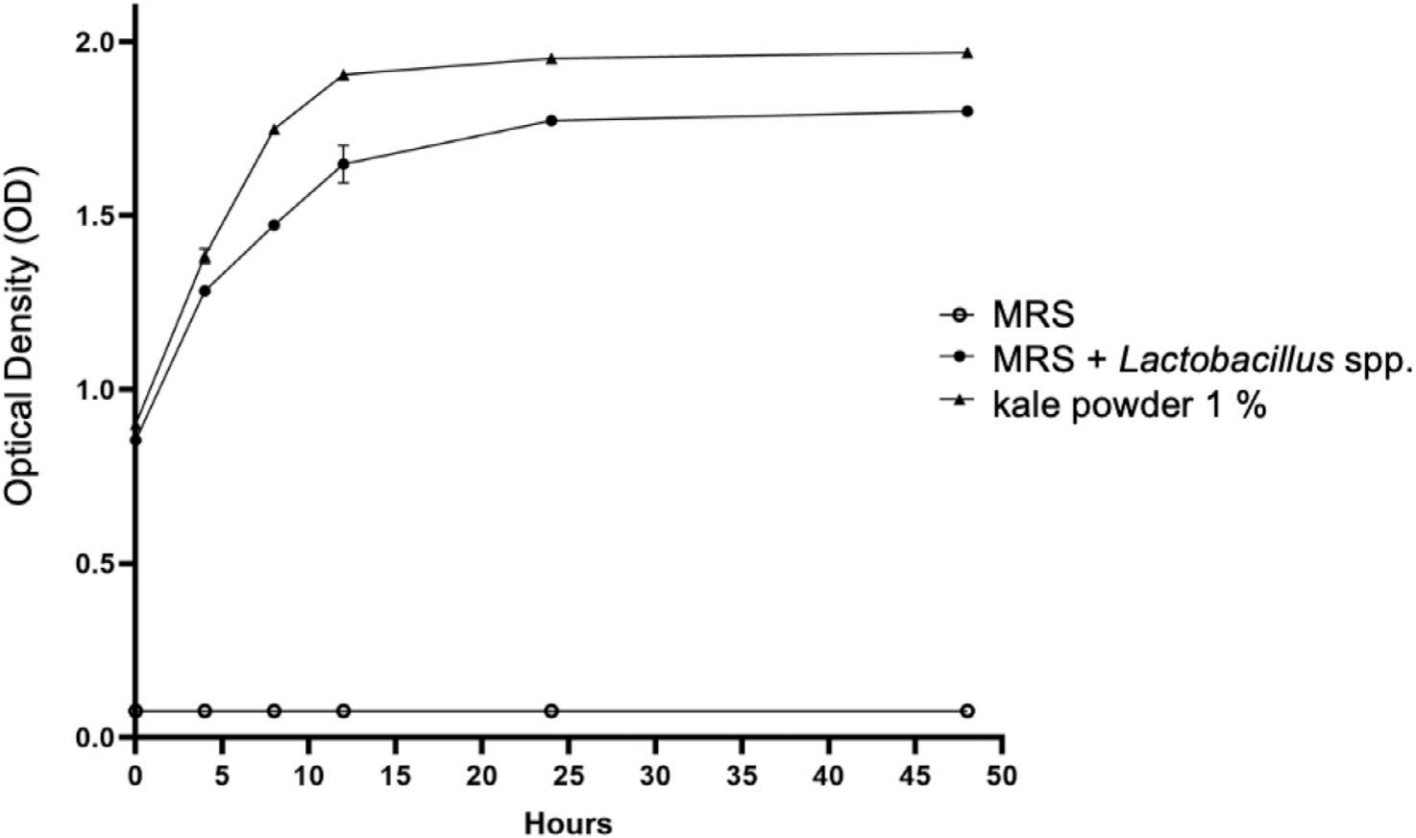
Growth kinetics of a Lactobacillus consortium incubated in MRS medium supplemented with 1% kale powder. The growth curve demonstrates that the addition of commercial kale powder significantly stimulates bacterial proliferation, achieving higher optical density (OD) values compared to the control (MRS medium alone). This enhancement suggests that the nutritional and prebiotic components present in kale serve as favorable substrates for the metabolic activity and growth of the probiotic consortium, supporting its potential application as a functional matrix in synbiotic formulations.

Kale, a nutrient‐dense leafy green, has gained attention as a potential prebiotic due to its rich profile of bioactive compounds. We hypothesized that its powder would enhance the growth and metabolic activity of a *Lactobacillus* spp. consortium, which we subsequently evaluated. The addition of 1% kale powder significantly enhanced the growth of *Lactobacillus* spp., as demonstrated in Figure 1. Notably, a pronounced increase in bacterial growth was observed starting at 5 h *post‐incubation;* in contrast to the control group, bacterial proliferation was restricted to the nutrients provided by the MRS medium alone. These findings indicate that specific components within kale powder act as effective substrates for the *Lactobacillus* spp. consortium, stimulating both growth and metabolic activity.

### 3.2 Development and Morphological Characterization of Synbiotic Encapsulated

**FIGURE 2:**
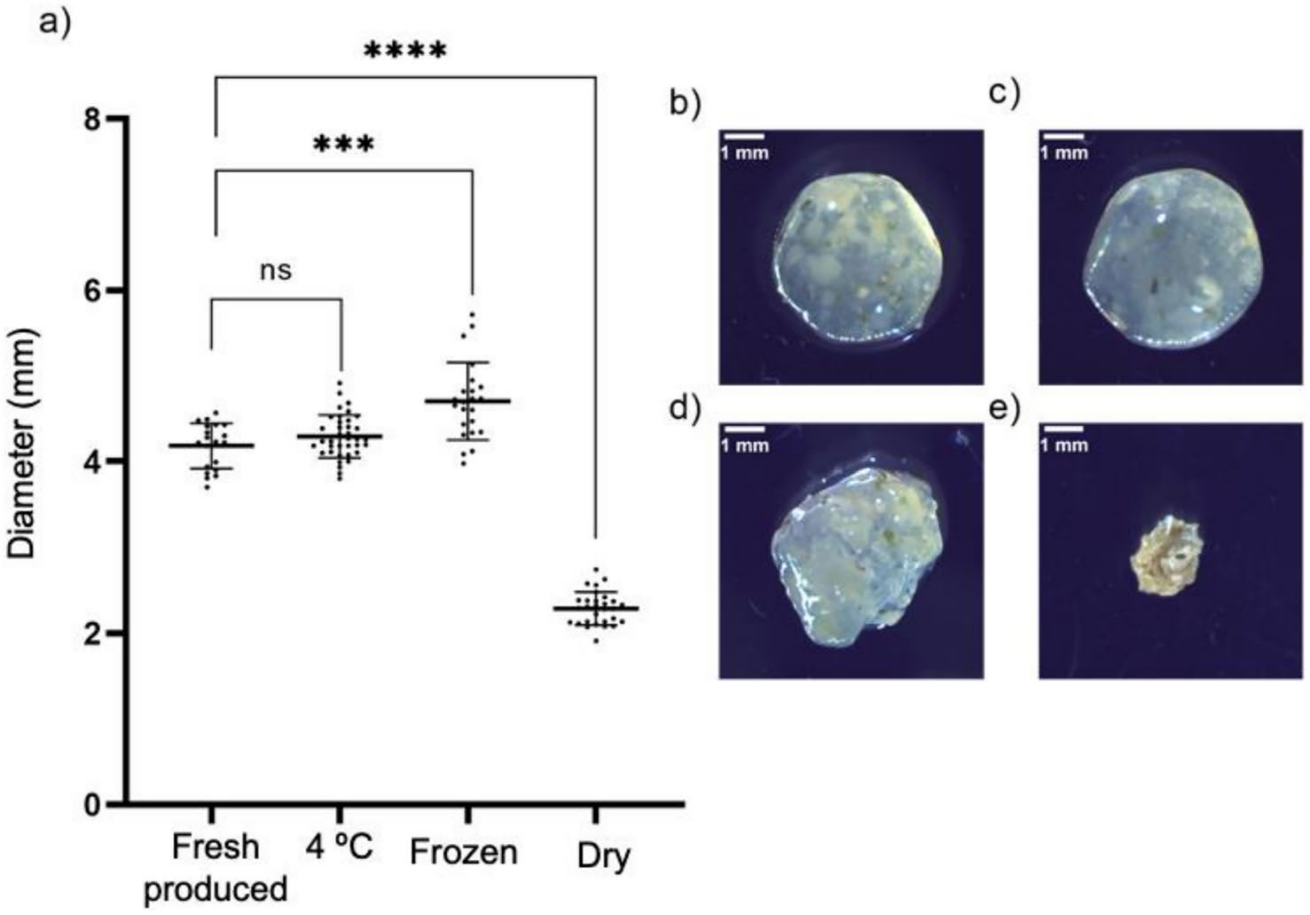
Morphological characterization of co‐encapsulated particles subjected to different temperature treatments. (a) Quantification of the average diameter of co‐encapsulated particles after storage at room temperature (freshly produced), refrigeration (4°C), freezing, and drying. A statistically significant reduction (*p* < 0.0001) in particle diameter was observed for dried samples, whereas freezing induced a slight increase compared to freshly produced controls. Representative images show: (b) freshly produced particles, (c) incubated at 4°C, (d) frozen, and (e) dried particles. These findings indicate that the drying process substantially alters particle morphology and size, while refrigeration and freezing better preserve structural integrity.

Co‐encapsulation is a complex process that involves multiple critical stages, including the selection of the matrix and the optimization of component concentrations. In this study, after extensive testing, a synergistic synbiotic was successfully co‐encapsulated using a 1.3% alginate‐based matrix combined with 1% kale powder and *Lactobacillus* spp. consortium. The morphological characteristics of the co‐encapsulated synbiotic were analyzed under different temperature conditions, as illustrated in Figure 2. Freshly produced particles exhibited a uniform size and structure, while those stored at 4°C maintained their integrity with minimal changes in diameter. In contrast, frozen particles showed a slight increase in diameter compared to the freshly produced control, likely due to the expansion of water during freezing. Markedly, dried particles significantly reduced diameter, reflecting the loss of moisture and structural compaction during the drying process. These findings highlight the influence of temperature treatments on the physical properties of the co‐encapsulated synbiotic.

### 3.3 Co‐Encapsulation Efficiency of Kale Powder and *Lactobacillus* spp. Consortium

**FIGURE 3:**
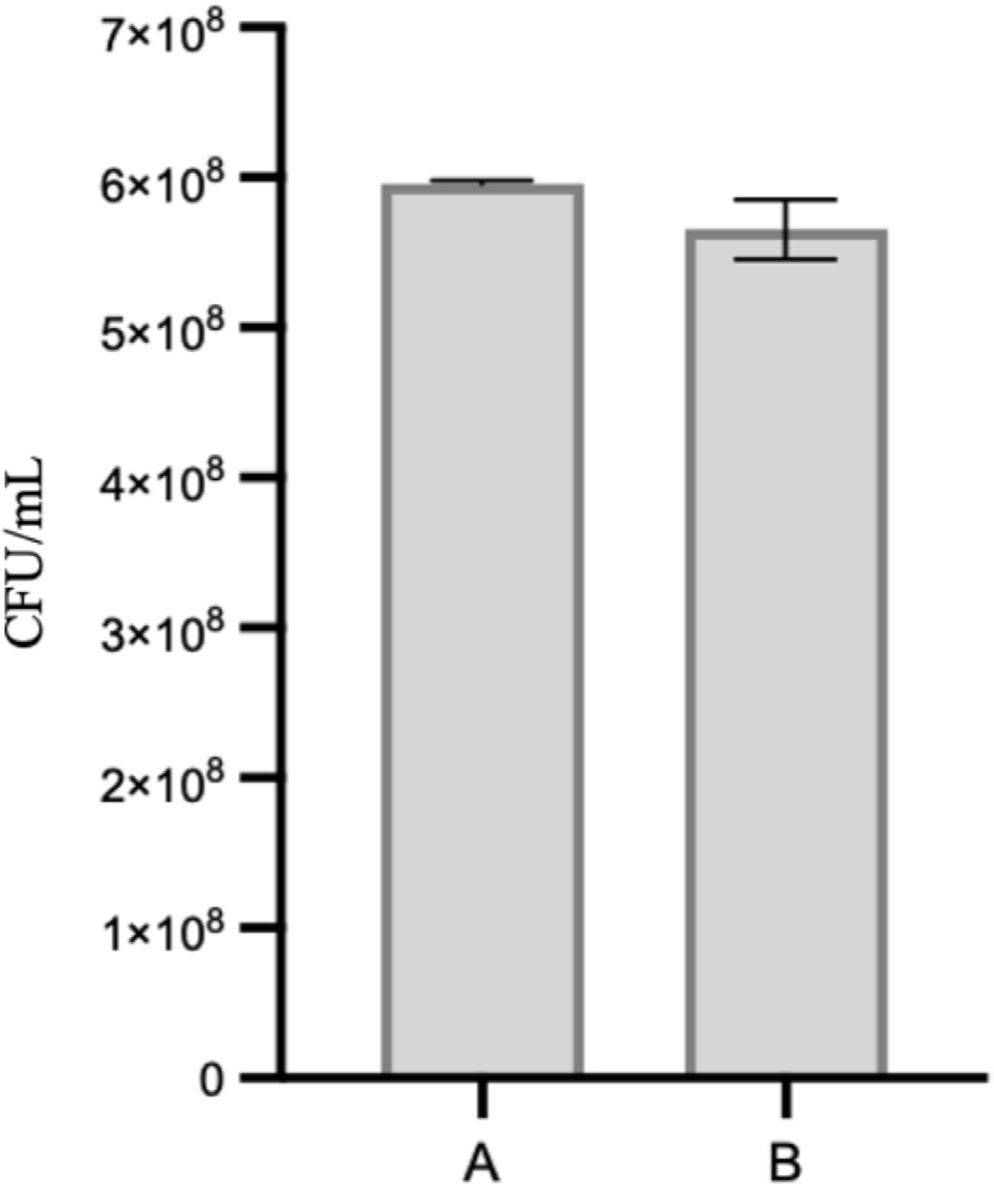
Encapsulation efficiency of the Lactobacillus consortium within a kale powder matrix. (a) Initial microbial load applied to the system prior to encapsulation (6 × 10^8^ CFU/mL); (b) recovered cell count after encapsulation (5.5 × 10⁸ CFU/mL). The calculated encapsulation efficiency was 93%, indicating high retention of viable cells within the kale matrix. This high yield suggests that the encapsulation method employed is effective in protecting and maintaining the viability of the probiotic consortium during formulation—a critical factor for its application in oral functional products.

Encapsulation efficiency and stability are critical parameters for synbiotic delivery systems. A 1.3% alginate concentration was selected for optimal wall integrity, demonstrating enhanced co‐encapsulation capacity for both the *Lactobacillus* consortium (initial concentration of 6 × 10^8^ CFU/mL) and kale compounds. The ionic gelation technique achieved 93% encapsulation efficiency, with 5.5 × 10^8^ CFU/mL recovered post‐encapsulation, confirming the alginate matrix's effectiveness in bacterial entrapment and viability (Figure 3).

### 3.4 Microstructural Characterization of Synbiotic Encapsulated

**FIGURE 4:**
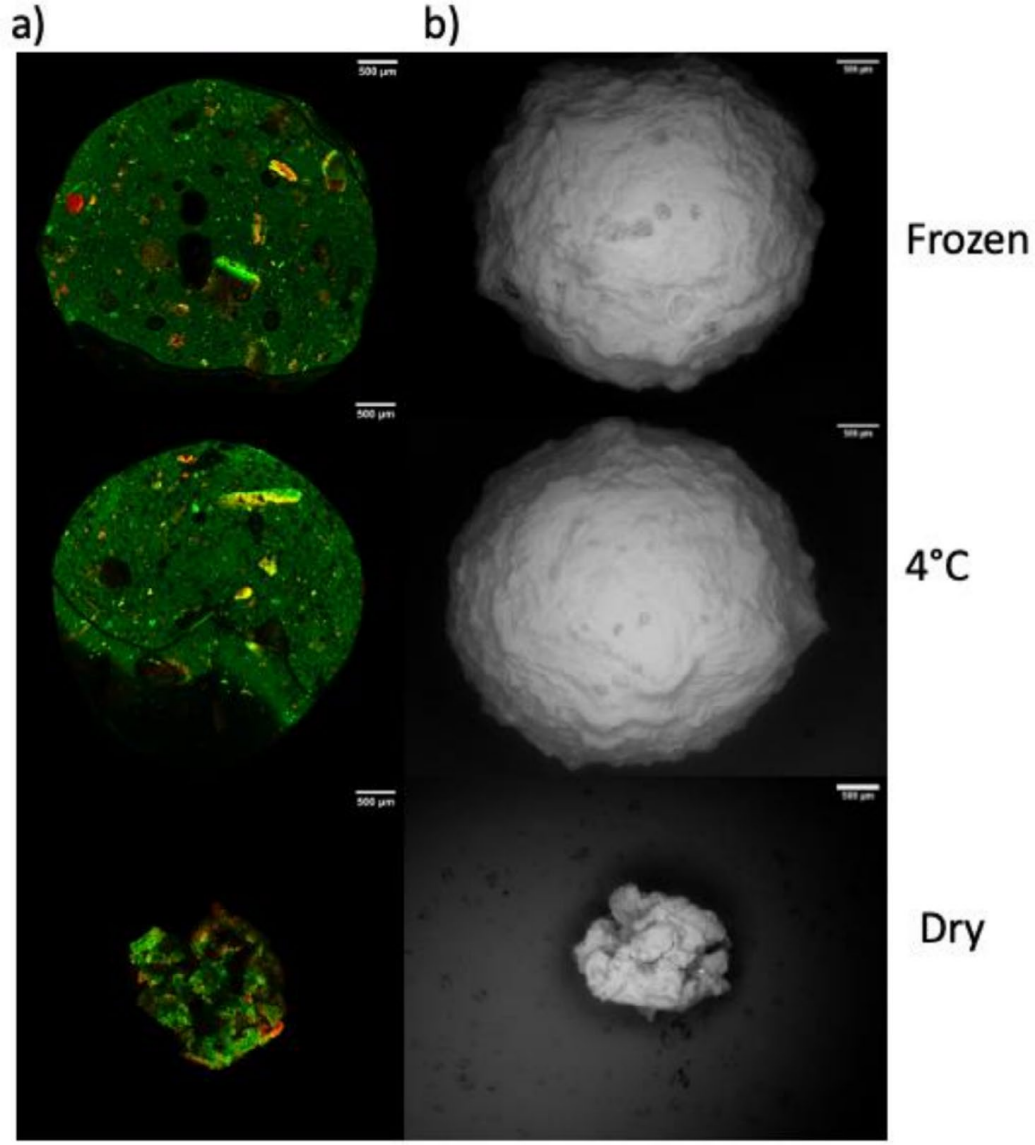
Microstructural analysis of co‐encapsulated particles using confocal and electron microscopy. (a) Representative confocal microscopy images of frozen, 4°C‐incubated, and dried particles. Strong green autofluorescence is evident, likely attributable to phenolic compounds derived from kale, which are uniformly distributed throughout the matrix. (b) Scanning electron microscopy images reveal that the surfaces of frozen and 4°C‐incubated particles remain intact and exhibit similar morphology, whereas dried particles display a rough, collapsed texture. These findings confirm that thermal treatments differentially affect particle structural integrity, with drying being the most disruptive.

To characterize the compounds within the co‐encapsulated system, microscopic analyses were performed under three conditions: stored at 4°C, after freezing, and following dehydration. Confocal microscopy revealed intense autofluorescence in the co‐encapsulated particles (Figure 4a), the green could correspond to several components such as pectins as reported by Wang et al. (2021), chitosan, as reported by Silva et al. (2022) or the alginate component, as demonstrated by Zhu et al. (2023) and Gómez‐Mascaraque et al. (2023). In analogous encapsulated systems additionally, the observed red fluorescence matched the spectral signature of phenolic compounds (García et al. 2022; Khatib et al. 2021), suggesting stable incorporation of kale polyphenols within the matrix. Electron microscopy analysis showed that the surface of the co‐encapsulated particles remained intact and exhibited similar morphology in both the frozen state and when stored at 4°C. In contrast, dehydrated particles displayed significant changes in the shape, size, and fluorescence intensity of the red component (Figure 4b). These alterations are likely due to the loss of liquid during drying, which may have increased the exposure of the red‐fluorescing component. Overall, the co‐encapsulated system demonstrated resilience to temperature variations, with minimal structural changes under cold storage conditions. However, dehydration led to notable morphological alterations, indicating that while the system is stable under refrigeration, it is more susceptible to structural changes during drying. These findings underscore the importance of optimizing storage conditions to preserve the integrity and functionality of the co‐encapsulated system.

### 3.5 Survival Analysis of *Lactobacillus* spp. Consortium After the Co‐Encapsulation With Kale Powder

**FIGURE 5:**
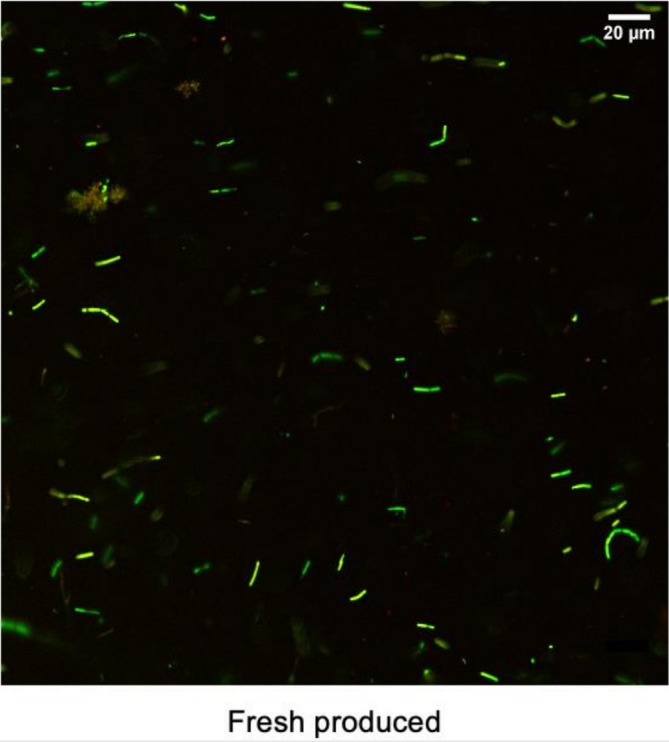
Survival and viability of the Lactobacillus consortium following the encapsulation process. Cell viability was assessed using dual fluorescent staining: acridine orange (green pseudocolor in micrographs) for viable cells and propidium iodide III (red or orange pseudocolor in micrographs) for nonviable cells. The confocal image shows freshly produced co‐encapsulates, revealing a high proportion of green‐stained cells (viable), indicating that the encapsulation process does not significantly compromise bacterial viability. This combined morphological and functional assessment confirms that co‐encapsulated particles retain high microbial viability—a key requirement for their efficacy as a synbiotic product.

The preservation of probiotic viability during storage is a critical factor in ensuring the efficacy of functional foods and supplements. To evaluate the effectiveness of co‐encapsulation in maintaining the survival of *Lactobacillus* spp. consortium, confocal microscopy analysis was employed. Cell viability was assessed using dual fluorescent staining: acridine orange (green pseudocolor), to identify viable cells and propidium III iodide (red or orange pseudocolor) to detect nonviable cells (Figure 5). The analysis was performed on freshly produced co‐encapsulates, providing insights into morphological integrity and cell viability. The results confirmed the successful survival of *Lactobacillus* spp. consortium within the co‐encapsulated system. These outcomes reinforce the potential of co‐encapsulation as a reliable strategy for enhancing stability, offering promising applications in the development of functional foods and dietary supplements.

### 3.6 Extraction, Quantification, and Growth Kinetics Analysis of Kale Polyphenols

**FIGURE 6:**
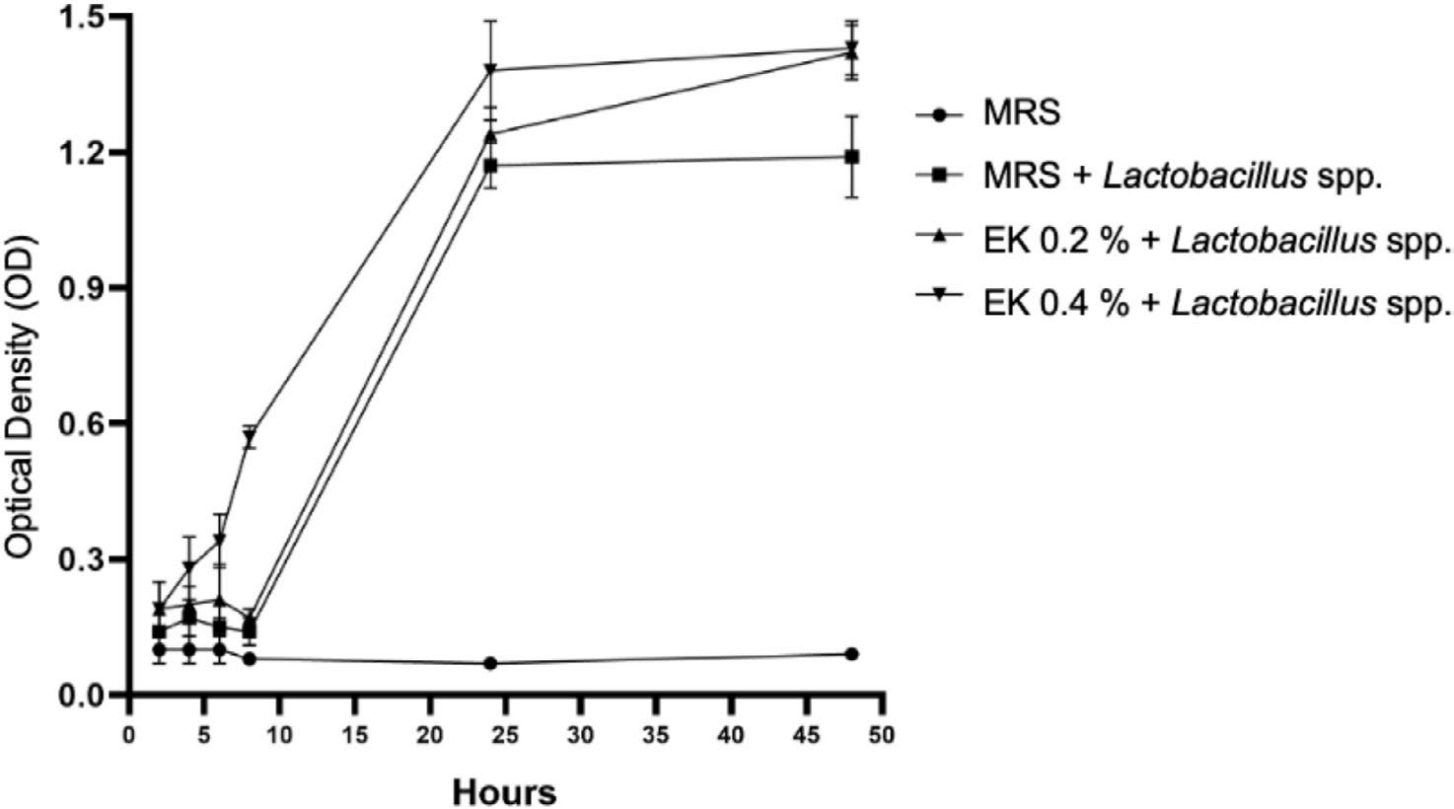
Growth kinetics of a Lactobacillus consortium in MRS medium supplemented with different concentrations of kale extract (EK). The effect of two kale extract concentrations (0.2% and 0.4%) on bacterial growth was evaluated and compared to the control (MRS medium alone). Both concentrations significantly stimulated the proliferation of the probiotic consortium, as evidenced by higher optical density (OD) values throughout the incubation period. The 0.4% concentration induced the greatest growth stimulation, reaching peak OD around 25 h of incubation. The 0.6% EK treatment was omitted from the graphical representation because it showed no statistically significant differences from the control, suggesting possible saturation or inhibitory effects at higher concentrations. These findings confirm that kale extract acts as a potent prebiotic for this consortium, with 0.4% being the optimal concentration for use in synbiotic formulations.

Microscopic analyses of co‐encapsulated systems have revealed the presence of polyphenols, as indicated by distinct red and green fluorescence, which likely contribute to the structural and functional integrity of the encapsulation matrix. These findings suggest that polyphenols may serve a dual role (Al‐Hamad and Raman 2017) protecting probiotics from environmental stress and (Andreasen et al. 2010) acting as potential substrates to support bacterial growth. To validate this hypothesis, we quantified kale‐derived polyphenols in aqueous extracts and evaluated their prebiotic potential by analyzing the growth kinetics of a *Lactobacillus* spp. consortium. The aqueous extract of kale was obtained with a yield of 15.84% (3.96 g) and resulting in a concentration of 810 mg GAE/100 g. To evaluate the potential of these polyphenols as substrates for bacterial growth, growth kinetics experiments were conducted using a *Lactobacillus* spp. consortium incubated in MRS medium supplemented with varying concentrations of kale extract (0.2%, 0.4%, and 0.6%). The results revealed that the medium supplemented with 0.4% total polyphenols promoted earlier growth of the *Lactobacillus* spp. consortium compared to the control, indicating enhanced metabolic activity. In contrast, the 0.2% concentration resulted in growth similar to the control, while the 0.6% concentration led to reduced bacterial growth, suggesting potential substrate inhibition at higher concentrations. These findings demonstrate that the *Lactobacillus* spp. consortium can utilize kale‐derived polyphenols as a metabolic substrate, with optimal growth observed at intermediate concentrations (Figure 6). This highlights the potential of kale polyphenols to serve as prebiotic compounds, supporting the growth and activity of probiotic bacteria.

## 4 Conclusion

In this study, a novel co‐encapsulation system combining *Lactobacillus* spp. with kale powder was successfully developed, proving to be an effective strategy for enhancing the viability and stability of probiotic bacteria. The addition of 1% kale powder, rich in phenolic compounds, demonstrated its prebiotic effect and its protective function *within* the capsule, achieving a 93% viability efficiency for the *Lactobacillus* consortium. Meanwhile, the capsule matrix itself was formed using a 1.3% sodium alginate solution, an optimal concentration for forming the gel beads that ensured structural integrity. Morphological and microstructural analyses confirmed the integrity of the alginate capsules under storage conditions at 4°C, while dehydration caused notable changes in their shape and size. These findings support the use of kale as a key functional food for designing synbiotics, opening new opportunities for its incorporation into food products to promote microbiota modulation and, consequently, intestinal health.

## 5 Discussion

This study demonstrates the significant potential of kale‐derived polyphenols as prebiotic compounds capable of enhancing the growth and stability of *Lactobacillus* spp., a key probiotic consortium. Growth kinetics experiments revealed that kale powder, particularly at a 1% concentration, significantly promoted bacterial proliferation, with a marked increase in growth observed after 5 h of incubation. This suggests that specific bioactive compounds in kale, such as polyphenols, act as effective substrates for *Lactobacillus* spp., supporting their metabolic activity and growth—a behavior previously reported by Gullón et al. (2015). Furthermore, optimal growth at intermediate polyphenol concentrations (0.4%) and reduced growth at higher concentrations (0.6%) underscore the importance of dosage in prebiotic applications, as excessive amounts may lead to substrate inhibition.

The successful co‐encapsulation of kale powder and the *Lactobacillus* spp. consortium using a 1% alginate matrix highlights the efficacy of this approach for creating stable and resilient synbiotic systems, consistent with findings by Cook et al. (2012) on alginate's protective role for probiotics. The encapsulation process achieved 93% efficiency, with the alginate matrix providing robust bacterial protection, evidenced by only a 13% reduction in viability after storage at 4°C. This cold‐stress resilience demonstrates the potential of alginate‐based encapsulation to preserve probiotic viability during refrigeration, a critical factor for functional food applications.

Morphological characterization of the co‐encapsulated system showed that particles maintained structural integrity under refrigeration and freezing, with minimal diameter changes. However, dehydration caused significant size reduction and morphological alterations, likely due to moisture loss (Yuan et al. 2022). These findings emphasize the need to optimize storage conditions to preserve the structural and functional integrity of co‐encapsulated systems.

Confocal and electron microscopy analyses confirmed the presence of polyphenols within the co‐encapsulated particles, with distinct autofluorescence attributed to phenolic compounds. This suggests that polyphenols not only contribute to the encapsulation matrix's structural stability but also play a role in protecting and maintaining bacterial viability.

Collectively, this study highlights the dual role of kale polyphenols as prebiotic substrates and protective agents in synbiotic systems, warranting further research for industrial food applications (Pandey et al. 2015). These results provide valuable insights for developing functional foods and dietary supplements that leverage kale's prebiotic potential and the protective capabilities of alginate encapsulation.

## Author Contributions

**Monica Villanueva‐Castañeda:** writing – original draft, visualization, methodology, investigation, formal analysis, conceptualization. **Mayra Antunez‐Mojica:** supervision, review and editing. **Daniel Tapia‐Maruri:** supervision, writing – review and editing. **Ollin Celeste Martínez Ramírez:** supervision, review and editing. **Raúl Dávila Delgado:** supervision, writing – review and editing. **América Ivette Barrera‐Molina:** writing – review and editing, supervision, resources, methodology, conceptualization.

## Conflicts of Interest

The authors declare no conflicts of interest.

## Data Availability

The data supporting the findings of this study are available from the corresponding author upon reasonable request. Microscopy images, microbial growth kinetics data, and morphological and microstructural characterization results can be provided by the corresponding author upon justified request.
